# The repertoire diversity of the *Plasmodium falciparum stevor* multigene family in complicated and uncomplicated malaria in India

**DOI:** 10.1186/1475-2875-11-S1-P125

**Published:** 2012-10-15

**Authors:** Surendra K Prajapati, Dolie D Laishram, Sanjib Mohanty, Jane M Carlton, Om P Singh

**Affiliations:** 1 Molecular Biology Division, National Institute of Malaria Research, New Delhi 110077, lndia; 2 Ispat General Hospital, Rourkela, Odisha 769011, India; 3 Center for Genomics and Systems Biology, Department of Biology, New York University, New York, NY 10003, USA

## Background

The deep vascular sequestration of parasitized erythrocytes is a central pathological event in falciparum malaria. Variant surface antigens are encoded mainly by three multi-copy gene families, namely, *var*, *stevor*, and *rifín. Var* is one of the most important families that plays a crucial role in antigenic variation and immune evasion. Clinical and epidemiological studies have shown that severe or complicated malaria is manifested in a limited number of patients. This indicates that a subset of these multigene families could be determinants in the manifestation of different malaria phenotypes. Recent studies have indicated the possible role of *stevor* (*sub-telomeric variable open read*), a multi-gene family, in erythrocyte invasion, antigenic variation and host cell modification, of infected erythrocytes. In this study, we describe the repertoire and diversity of members of the *stevor* multigene family in patients with complicated and uncomplicated malaria in India.

## Materials and methods

*Plasmodium falciparum* complicated isolates (n=8) from Odisha and uncomplicated isolates (n=7) from Assam, Madhya Pradesh and Goa were collected. Members of the *stevor* multigene family were amplified using degenerate PCR primers. Amplified PCR products were cloned and a total of 35 clones per cloning experiment were sequenced. A maximum likelihood phylogeny was constructed in order to understand the genetic repertoire of members of the *stevor* multigene family in severe and non-severe isolates and extent of *stevor* repertoire in Indian isolates.

## Results

A range of 21-31 unique sequences was obtained out of 35 clones sequenced for each of the 15 isolates. Nucleotide diversity analysis shows extensive genetic polymorphism that supports the hyper-variability nature of *stevor* multigene family in field isolates. The repertoire and diversity of the *stevor* multigene family varied between all four geographical regions of the Indian subcontinent. Phylogenetic tree analysis showed clustering of sequences from complicated isolates, and suggests that the *stevor* genetic repertoire is less diverse in comparison to uncomplicated isolates.

## Conclusions

This study suggests an extensive genetic diversity of *stevor* in Indian *P. falciparum* isolates, however the genetic repertoire from complicated cases was less diverse. The high degree of *stevor* diversity has important implications for the design of effective anti-malaria control measures.

